# Pharyngeal Airway and Craniocervical Angle among Different Skeletal Patterns

**DOI:** 10.1155/2021/5536464

**Published:** 2021-07-15

**Authors:** Szu-Yu Hsiao, Ying-An Chen, Chun-Chan Ting

**Affiliations:** ^1^School of Dental Medicine, Kaohsiung Medical University, Kaohsiung, Taiwan; ^2^Department of Dentistry for Child and Special Needs, Kaohsiung Medical University Hospital, Kaohsiung, Taiwan; ^3^Dental Department, Zhongxiao Branch of Taipei City Hospital, Taipei, Taiwan; ^4^School of Dentistry & Institute of Oral Medicine, National Cheng Kung University, Tainan, Taiwan; ^5^Department of Stomatology, National Cheng Kung University Hospital, Tainan, Taiwan

## Abstract

**Purpose:**

The aim of the present study was to investigate the pharyngeal airway dimensions and their correlations among the craniocervical angle and skeletal patterns.

**Materials and Methods:**

Cephalometric radiographs were obtained from 300 patients (≥15 years of age), of whom 150 were male patients and 150 were female patients. The patients were divided into three groups according to their skeletal patterns. The following dimensions were measured: NP: nasopharyngeal airway; PS: shortest distance from the soft palate to the pharyngeal wall; MP: Me-Go line intersecting the pharyngeal airway; TS: shortest distance from posterior tongue to pharyngeal wall; LP: laryngopharyngeal airway; UE length: shortest distance from the uvula to the epiglottis; PW: width of soft palate; PL: length of soft palate; ANB angle; palatal angle; and craniocervical angle. Paired *t*-test, one-way analysis of variance (ANOVA), and Pearson correlation were applied for statistical analysis. The null hypothesis was that there were no differences among skeletal patterns in terms of pharyngeal airway dimensions.

**Results:**

The C4C2-SN angle of the Class II pattern (108.1°) was significantly greater than that of the Class III pattern (104.4°). The Class II PL was significantly longer than the Class III PL in the all patients and female patients groups. The ANB angle exhibited moderate positive correlation with palatal angle (*r*: 0.462) and moderate negative correlation with TS (*r*: -0.400) and MP (*r*: -0.415) length. No significant differences were found in vertical hyoid lengths among all skeletal patterns. Class III (PS, TS, and MP) lengths were significantly greater than Class I and Class II in the all patients group. Regarding the LP length, no significant difference was found in the all patients group. Therefore, the null hypothesis was rejected.

**Conclusion:**

Class III had significantly greater pharyngeal airway dimensions (PS, TS, and MP) than Class I and Class II. In all skeletal patterns, NP length was moderately correlated with the palatal angle. The PS was weakly negatively correlated with the ANB and PL. The TS and MP were moderately negatively correlated with the ANB angle.

## 1. Introduction

The pharynx is crucial to respiration, deglutition, and vocalization. The pharynx is a cone-shaped passage that links the oral and nasal cavities to the esophagus and the trachea. The pharynx is primarily composed of the nasopharynx, oropharynx, and laryngopharynx. The nasopharynx and oropharynx are divided by the posterior soft palate of the upper jaw, whereas the oropharynx and laryngopharynx are divided by the tip of the epiglottis. The nasopharynx is the uppermost part of the pharynx, which comprises a cavity above the soft palate and posterior nasal cavity, where the nasal passages, inner ear channel, and pharynx meet. Handelman and Osborne [1] reported that the growth of the nasopharynx diameter continues to approximately 13 years of age. Vilella et al. [2] reported that nasopharynx and adenoidal development growth peak may be reached 15 years of age.

Regarding maxillary development, Nanda [3] suggested that maxillary growth is minimal beyond the age of 12 years. Bae et al. [4] reported that the growth peak of the mandibular corpus occurs between the ages of 13 and 15 years and that males demonstrate considerably greater growth than females. Taylor et al. [5] investigated the pattern of bony and soft tissue growth of the oropharynx. They found that posterior nasal spine to pharyngeal wall and posterior soft palate to pharyngeal wall increased accelerate change (6–9 years and 12–15 years) and two periods of quiescence (9–12 years and 15–18 years) were identified. Therefore, pharynx airway development is nearly complete at the age of 15 years. This present study investigated skeletal patterns and pharyngeal airways with respect to the morphologies of the maxilla, soft palate, tongue, mandible, and hyoid bone.

## 2. Materials and Methods

**Figure 1:**
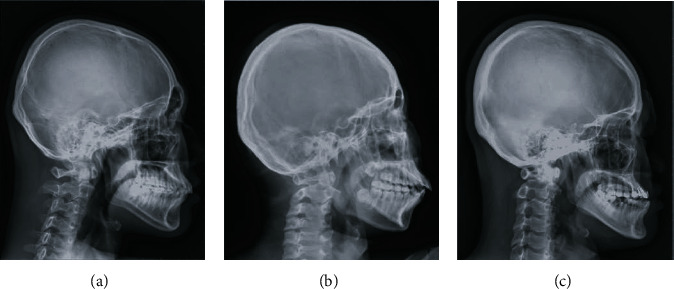
Skeletal patterns from right to left: (a) Class I, (b) Class II, and (c) Class III.

Cephalometric radiographs (Department of Dentistry, Kaohsiung Medical University Hospital) were obtained from 300 patients (≥15 years of age), of whom 150 were male patients and 150 were female patients. The patients were divided into three groups (Figure 1) according to their skeletal patterns (specifically, the A point–nasion–B point (ANB) angle): Class I (0° < ANB < 4°), Class II (ANB ≥ 4°), and Class III (ANB ≤ 0°). Each group consisted of 100 patients, with a sex ratio of 50 male patients/50 female patients. The exclusion criteria were as follows: (1) patients with craniofacial symptoms or deformity, (2) patients who had experienced craniofacial bone surgeries, and (3) patients who had a history of maxillofacial trauma.

**Figure 2:**
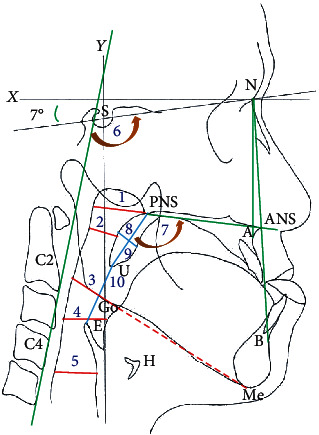
Cephalometric landmarks and linear measurements. Landmarks: nasion (N); sella (S); anterior nasal spine (ANS); point A; posterior nasal spine (PNS); point B; menton (Me); tip of uvula (U); inferoanterior point on the fourth cervical (C4); inferoanterior point on the second cervical (C2); most superior and anterior point on the hyoid bone (H); most superior point on the epiglottis (E); and gonion (Go). The *X*-axis was constructed by drawing a line through the N, 7° above the SN line; the *Y*-axis was constructed by drawing a line through S, perpendicular to the *X*-axis. Red line: 1: NP; 2: PS; 3: MP; 4: TS; 5: LP. Blue line: 8: PW; 9: PL; 10: UE. Angle measurement: 6: C2C4-SN; 7: palatal angle.

The following landmarks (Figure 2) were identified on each cephalogram: nasion (N); sella (S); anterior nasal spine (ANS); point A; posterior nasal spine (PNS); point B; menton (Me); tip of uvula (U); inferoanterior point on the fourth cervical (C4); inferoanterior point on the second cervical (C2); most superior and anterior point on the hyoid bone (H); most superior point on the epiglottis (E); and gonion (Go). The *X*-axis was constructed by drawing a line through the nasion, 7° above the SN line; the *Y*-axis was constructed by drawing a line through S, perpendicular to the *X*-axis. Linear and angular measurements included the following: NP: nasopharyngeal airway (ANS-PNS plane intersecting the pharyngeal wall); PS: shortest distance from the soft palate to the pharyngeal wall; MP: Me-Go line intersecting the pharyngeal airway; TS: shortest distance from posterior tongue to pharyngeal wall; LP: laryngopharyngeal airway (horizontal plane through C4, intersecting the pharyngeal wall); UE: shortest distance from the uvula to the epiglottis; PW: width of soft palate; PL: length of soft palate; ANB angle; palatal angle; C2C4-SN angle: angle between the C4C2 line and SN line; HH: horizontal position of hyoid; HV: vertical position of hyoid.

The data were processed by using IBM SPSS 20 (SPSS Inc., Chicago, IL, USA). In the cephalometric analysis, landmarks for soft and hard tissues were identified according to the results of paired *t*-test analyses of the male and female patients in each group. Intergroup comparison analysis was conducted with one-way analysis of variance (ANOVA), and post hoc comparisons were performed by using Tukey's honest significant difference test. Correlations between variables were examined by using Pearson correlation analysis. Strengths of correlation were described for the absolute value of the ratio of the compared variables: very weak (0–0.19), weak (0.20–0.39), moderate (0.40–0.59), strong (0.60–0.79), and very strong (0.80–1.0). The level of significance was set as *p* < 0.05. The null hypothesis was that there were no differences among skeletal patterns in terms of pharyngeal airway dimensions. This was a retrospective study, approved by the human investigation review committee at the Kaohsiung Medical University Hospital.

## 3. Results

As shown in Table 1, age was not significantly different among the skeletal patterns. The C4C2-SN angle of the Class II pattern (108.1°) was significantly greater than that of the Class III pattern (104.4°). In terms of sex comparison, the C4C2-SN angle in female patients with the Class I pattern was significantly greater than in their male counterparts. The palatal angle of the Class II pattern (127.4°) was significantly greater than that of the Class I pattern (124.3°), which was in turn significantly greater than that of the Class III pattern (120.4°). Furthermore, palatal angles in female patients with Class I and Class II patterns were significantly greater than in their male counterparts. As shown in Table 2, changes in the female patient group were similar to changes observed for all patients (Table 1). In contrast, male patients (Table 3) showed no differences in the C2C4-SN angle among all skeletal patterns.

The PW did not differ in the all patients group (Table 1), female patients group (Table 2), or male patients group (Table 3). The Class II PL was significantly longer than the Class III PL in the all patients and female patients groups. The PL did not differ in the male patient group. The Class II UE length (24.3 mm) was significantly longer than the Class III UE length (21.3 mm) in female patients. The UE length did not differ in the all patients and male patients groups. Class III and Class I horizontal hyoid lengths (19.1 mm and 16.4 mm, respectively) were significantly greater than the corresponding Class II lengths (12.2 mm); horizontal hyoid lengths among male patients were significantly greater than those of female patients. No significant differences were found in vertical hyoid lengths among all skeletal patterns; however, male patients exhibited significantly greater vertical hyoid lengths, compared with female patients.

Comparison of the various pharyngeal airway lengths revealed the following results: Class II NP length (25.2 mm and 25.5 mm) was significantly greater than Class I NP length in the all patients (24 mm) and Class I NP female patients (24 mm) groups, but no significant difference was observed in the male patients group. Class III PS length was significantly greater than Class I and Class II PS length in the all patients group (Class III: 12.7 mm, Class I: 11 mm, and Class II: 9.9 mm), female patients group (Class III: 12.2 mm, Class I: 10.5 mm, and Class II: 10.3 mm), and male patients group (Class III: 13.1 mm, Class I: 11.4 mm, and Class II: 9.6 mm). Class III TS length was significantly greater than Class I and Class II TS length in the all patients group (Class III: 14.5 mm, Class I: 12.4 mm, and Class: 11.3 mm), female patients group (Class III: 13.5 mm, Class I: 12 mm, and Class II: 11.2 mm), and male patients group (Class III: 15.6 mm, Class I: 12.7 mm, and Class II: 11.5 mm). Class III MP length was significantly greater than Class I and Class II MP length in the all patients group (Class III: 16.1 mm, Class I: 13.8 mm, and Class II: 12.3 mm), female patients group (Class III: 15.4 mm, Class I: 13.2 mm, and Class II: 12.7 mm), and male patients group (Class III: 16.9 mm, Class I: 14.4 mm, and Class II: 11.9 mm). Regarding the LP length, no significant difference was found in the all patients group, but the value of the male patients group was significantly greater than that of the female patients group in each class. In the male patients group, Class III LP length (19.5 mm) was significantly greater than Class II (17.5 mm) and Class I (17.4 mm) LP lengths. Therefore, the null hypothesis was rejected.

**Figure 3:**
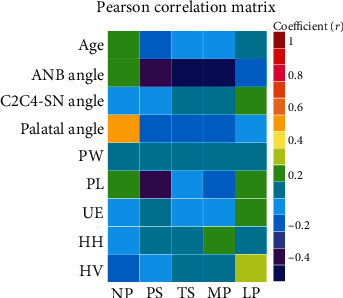
Pharyngeal airway in the Pearson correlation matrix. Absolute value of correlation ratio: very weak (0-0.19), weak (0.20-0.39), moderate (0.40-0.59), strong (0.60-0.79), and very strong (0.80-1.0). White circle: statistically significant, *p* < 0.05.

Table 4 and Figure 3 showed the results of Pearson's correlation test in the all patients group. The ANB angle exhibited moderate positive correlation with palatal angle (*r*: 0.462) and moderate negative correlation with TS (*r*: -0.400) and MP (*r*: -0.415) length. Palatal angle exhibited moderate positive correlation with NP (*r*: 0.439) length. Horizontal distance of hyoid bone exhibited strong negative correlation with C2C4-SN angle (*r*: -0.696) and moderate negative correlation with palatal angle (*r*: -0.491).

## 4. Discussion

The nasopharynx is the uppermost part of the pharynx. Deepthi et al. [6] evaluated the airway in the Class I and Class II skeletal pattern. They found a strong association between the airway and skeletal pattern showing a reduced nasopharyngeal airway in Class II patients with a high ANB angle compared to Class I. In the present study, NP of Class II was significantly greater than Class I in the all patients and female patients groups. Moreover, Pearson correlation analysis indicated that, of all the pharyngeal airway lengths, NP had a moderate positive correlation with the palatal angle. Thus, it can be inferred that the palatal angle is an optimal predictor of NP length because the palatal angle increases as NP lengthens. Conversely, NP length was not correlated with the C2C4-SN angle, the degree of ANB, width and length of soft palate, or the horizontal and vertical hyoid positions. Hoffstein et al. [7] examined the flow-volume curves in snoring patients with and without obstructive sleep apnea. They found no significant difference in the midvital capacity flow ratio between the two groups. Therefore, we inferred that the correlation between NP length and occurrence of obstructive sleep apnea was not significant.

Abu Allhaija and Al-Khateeb [8] reported no significant difference in the PW and PL among different anteroposterior skeletal patterns. Muto et al. [9] measured anteroposterior diameter of the pharyngeal airway space in patients with mandibular retrognathia and prognathia, and normal subjects. They reported no difference in PW but mandibular retrognathia was significantly greater than Class I and Class III in PL. In our study, PW also showed no difference in all patients, female patients, or male patients groups. The PL of Class II was significantly greater than Class III in the all patients and female patients groups. Among different skeletal patterns, PL showed no difference in the male patients group. Abu Allhaija and Al-Khateeb [8] also reported no sex differences at the PW and PL among skeletal patterns. In our study, we found that PW of the males group had greater than the females group in Class II and Class III. The PL of the males group had greater than the females group in Class I and Class III. In terms of hyoid position, a more inferior position of the hyoid could significantly increase the dimensions of PW and PL. An anterior position of the hyoid also could significantly increase PW, but not PL.

Moreover, we found that there was no significant correlation between PW and PL. In Pearson's correlation analysis, PW had no effect on the pharyngeal airway dimensions. Therefore, PW was not a risk factor leading to occurrence of obstructive sleep apnea. Muto et al. [9] found that PL was a significant negative correlation with PS in the normal mandible, mandibular retrognathism, and all patients groups. In our study, PL was significantly and positively correlated with ANB angle and HV, indicating that downward growth of the hyoid bone increases the PL. PL was also a significant negative correlation with PS in all patients groups. In terms of the correlation of the ANB angle with features of palatal-related anatomy, all features exhibited a significant correlation with the ANB angle. In Pearson's correlation analysis, PS airway was significantly and negatively correlated with ANB. Therefore, Class III had the shortest palatal length and greatest PS length, whereas Class II had the greatest PL and shortest PS length. However, PL was only weakly negatively correlated with PS length (PL increased as PS length decreased). Therefore, we could not infer that PL was strongly correlated with PS length.

Muto et al. [9] reported that the palatal angle of Class II was significantly greater than Class I and Class I was significantly greater than Class III. Our finding was similar to the report of Muto et al. [9] The palatal angle was strongly positively correlated with the ANB angle and C4C2 angle; however, we found that the palatal angle showed no significant correlation with PW and PL. Moreover, the palatal angle was a significant correlation with pharyngeal airway dimensions except LP. The incremental palatal angle increased the NP but decreased PS, TS, and MP.

The UE length revealed a trend where a more inferior position of the hyoid caused greater UE length. In our study, UE presented no difference in the male patients and all patients groups. In the female patients group, UE of Class II was significantly greater than Class III. The present study also found a significant and positive correlation between UE and hyoid bone growth, indicating that downward growth of the hyoid bone increases UE. This suggests that the epiglottis also grows downward to increase the length of UE. Therefore, UE cannot predict the Class I and Class II patients that are most likely to exhibit obstruction of the pharyngeal airway.

The ANB angle was negatively correlated with HH. Therefore, a greater ANB angle corresponded to a shorter horizontal hyoid length and a relatively retracted hyoid bone position. The role of the C4C2 angle was similar to that of the ANB angle; however, the C4C2 angle was even more strongly correlated with the hyoid bone position (-0.696), such that the cervical spine position was closely related to the hyoid bone position (a retracted hyoid bone position corresponds to a greater C4C2 angle). This may be attributed to the physiological regulation of respiration, because a retracted hyoid bone can constrict the respiratory tract, compelling the C4C2 angle to increase to maintain smooth breathing. This is typically achieved by raising the head slightly.

A reduction or increase of the oropharyngeal cavity can be induced by tongue retraction or tongue protrusion. Because the base of the tongue is connected with the hyoid bone, the pharyngeal-airway muscle groups are connected to the soft palate and tongue [10]. Adamidis and Spyropoulos [11] compared the hyoid bone positions in Class I and Class III and found that the hyoid bone position in Class III was set relatively forward. Yamaoka et al. [12] found that the tongue root in Class II was relatively retracted, compared with that in Class III. Battagel et al. [13] examined patients with obstructive sleep apnea and noticed that they exhibited Class II occlusion, with relatively retracted hyoid bone positions, which resulted in a narrower pharyngeal airway. These studies suggest that the hyoid bones of people with Class II features are relatively retracted, whereas the hyoid bones of people with Class III features are relatively forward. Our finding was similar to previous reports. The hyoid bone in Class III is set significantly forward, compared with that of Class I and Class II; thus, its position is negatively correlated with the ANB angle. However, the horizontal hyoid position was only weakly correlated with MP length and showed no correlation with PS, TS, and LP. Mortazavi et al. [14] reported that hyoid bone is positioned more superior and posterior in females than males and its location differs among different skeletal classes. Our study was similar to the report of Mortazavi et al. [14]

In a study of the relationship between the pharyngeal airway and skeletal patterns, Muto et al. [9] found the pharyngeal airway of Class III to possess the greatest space, followed by Class I and Class II. In our study, TS and MP were between the tongue and the pharyngeal airway. We found that both TS and MP lengths were significantly shorter in Class II, which echoes the findings of Muto et al. [9] The present study found the TS and MP lengths to be more significantly correlated with the ANB angle than with the C2C4 angle. That is, the TS and MP lengths were more related to the skeletal pattern than the position of the cervical spine. Furthermore, TS and MP lengths were not significantly correlated with palatal-related anatomy (PW, PL, and UE). Of particular interest is the hyoid bone position, whose correlation with the MP was weak and with TS was nonsignificant. Thus, the hyoid bone position cannot be used to estimate TS and MP lengths. In terms of LP length, which represents the distance from C4 to the front tracheal wall, no significant difference was observed among the skeletal patterns. Moreover, Pearson's correlation analysis revealed a weak correlation between LP length and the other factors, indicating that the influence of anatomical structure on LP length is minimal. The limitation of the present study is used to the two-dimensional (2D) cephalograph to represent the complex 3D pharyngeal structure. The major limitations of 2D cephalometric analysis are the lack of information of cross-sectional area and real pharyngeal volume (3D).

## 5. Conclusion

Among the pharyngeal airways of skeletal patterns, Class III had significantly greater pharyngeal airway dimensions (PS, TS, and MP) than Class I and Class II. Class II had the largest NP than Class I and Class III. The C4C2-SN angle of Class II was significantly greater than that of Class III. The C4C2-SN angle exhibited no significant correlation with pharyngeal airway dimensions (NP, PS, TS, and MP).

## Figures and Tables

**Table 1:** Patient characteristics in the skeletal classification (one-way ANOVA).

Variables	Class I (F = 50; M = 50)			Class II (F = 50; M = 50)			Class III (F = 50; M = 50)			Intergroup comparison		
	Mean	SD		Mean	SD		Mean	SD		*p* value		Significant
Age	23.9	6.20	─	24.2	5.95	─	23.4	5.97	─	0.671	─	
ANB	2.2	0.92	─	6.2	1.90	─	-2.9	2.73	─	<0.001	†	Class II > Class I > Class III
C2C4	106.5	7.42	∗(F > M)	108.1	6.49	─	104.4	8.16	─	0.002	†	Class II > Class III
Palate	124.3	6.02	∗(F > M)	127.4	6.45	∗(F > M)	120.4	7.72	─	<0.001	†	Class II > Class I > Class III
PW	8.7	1.87	─	8.6	1.94	∗(M > F)	9.2	2.02	∗(M > F)	0.073	─	
PL	35.5	4.17	∗(M > F)	36.6	4.50	─	34.4	4.01	∗(M > F)	0.001	†	Class II > Class III
UE	27.9	20.77	∗(M > F)	27.3	6.10	∗(M > F)	25.7	7.74	∗(M > F)	0.471	─	
Pharyngeal airway												
NP	24.0	3.46	─	25.2	3.14	─	24.2	3.60	─	0.024	†	Class II > Class I
PS	11.0	3.08	─	9.9	2.91	─	12.7	3.62	─	<0.001	†	Class III > Class I, Class III > Class II
TS	12.4	3.37	─	11.3	3.27	─	14.5	4.39	∗(M > F)	<0.001	†	Class III > Class I, Class III > Class II
MP	13.8	3.70	─	12.3	3.56	─	16.1	4.86	─	<0.001	†	Class III > Class I > Class II
LP	16.4	3.51	∗(M > F)	16.7	3.30	∗(M > F)	17.5	4.09	∗(M > F)	0.079	─	
Hyoid												
HH	16.4	9.13	∗(M > F)	12.2	8.06	∗(M > F)	19.1	9.95	∗(M > F)	<0.001	†	Class III > Class II, Class I > Class II
HV	122.7	10.01	∗(M > F)	122.6	10.66	∗(M > F)	121.7	10.63	∗(M > F)	0.762	─	

F: female; M: male; ─: not significant. ∗: intergender comparison: statistically significant, *p* < 0.05. †: intergroup comparison: statistically significant, *p* < 0.05.

**Table 2:** The characteristics of female patients in the skeletal classification (one-way ANOVA).

Variables	Class I (*n* = 50)		Class II (*n* = 50)		Class III (*n* = 50)		Intergroup comparison		
	Mean	SD	Mean	SD	Mean	SD	*p* value		Significant
Age	23.2	5.41	23.3	4.87	23.3	5.74	0.998	─	
ANB	2.3	0.95	6.3	2.12	-2.7	2.63	<0.001	∗	Class II > Class I > Class III
C2C4	108.6	6.57	108.4	6.84	104.4	8.38	0.006	∗	Class I > Class III; Class II > Class III
Palate	125.9	5.75	128.9	6.07	121.6	6.88	<0.001	∗	Class II > Class I > Class III
PW	8.5	1.67	7.9	1.69	8.5	1.40	0.121	─	
PL	34.4	3.65	36.3	4.76	33.5	3.61	0.003	∗	Class II > Class I; Class II > Class III
UE	23.3	5.66	24.3	4.94	21.3	5.05	0.015	∗	Class II > Class III
Pharyngeal airway									
NSP	24.0	3.50	25.5	2.73	24.1	3.48	0.040	∗	Class II > Class I
PS	10.5	3.05	10.3	2.86	12.2	3.62	0.006	∗	Class III > Class I; Class III > Class II
TS	12.0	3.08	11.2	2.57	13.5	3.53	0.001	∗	Class III > Class II
MP	13.2	3.35	12.7	3.28	15.4	4.29	0.001	∗	Class III > Class I; Class III > Class II
LGP	15.5	3.21	15.9	2.66	15.5	3.29	0.809	─	
Hyoid									
HH	12.9	7.41	9.6	6.17	17.3	8.90	<0.001	∗	Class III > Class I; Class III > Class II
HV	116.2	7.44	114.9	6.07	113.3	6.02	0.098	─	

*n*: number of patient. ∗: statistically significant, *p* < 0.05; ─: not significant.

**Table 3:** The characteristics of male patients in the skeletal classification (one-way ANOVA).

Variables	Class I (*n* = 50)		Class II (*n* = 50)		Class III (*n* = 50)		Intergroup comparison		
	Mean	SD	Mean	SD	Mean	SD	*p* value		Significant
Age	24.6	6.89	25.1	6.79	23.6	6.25	0.505	─	
ANB	2.1	0.90	6.2	1.66	-3.2	2.83	<0.001	∗	Class II > Class I > Class III
C2C4	104.5	7.71	107.7	6.17	104.3	8.01	0.062	─	
Palate	122.7	5.90	125.8	6.50	119.3	8.38	<0.001	∗	Class I > Class III; Class II > Class III
PW	8.9	2.05	9.2	1.99	9.8	2.35	0.107	─	
PL	36.7	4.36	36.8	4.25	35.2	4.25	0.105	─	
UE	32.6	28.22	30.2	5.76	30.1	7.52	0.718	─	
Pharyngeal airway									
NSP	23.9	3.47	24.9	3.51	24.3	3.75	0.375	─	
PS	11.4	3.09	9.6	2.94	13.1	3.60	<0.001	∗	Class III > Class I > Class II
TS	12.7	3.63	11.5	3.87	15.6	4.93	<0.001	∗	Class III > Class I; Class III > Class II
MP	14.4	3.96	11.9	3.81	16.9	5.31	<0.001	∗	Class III > Class I > Class II
LGP	17.4	3.59	17.5	3.69	19.5	3.84	0.005	∗	Class III > Class I; Class III > Class II
Hyoid									
HH	19.8	9.44	14.9	8.90	20.8	10.69	0.005	∗	Class III > Class II; Class II > Class I
HV	129.2	7.75	130.3	8.48	130.1	6.98	0.766	─	

*n*: number of patient. ∗: statistically significant, *p* < 0.05; ─: not significant.

**Table 4:** Pearson correlation (*r*) test for craniofacial angles and linear distances in all patients.

Variables	ANB angle	C2C4-SN angle	Palatal angle	PW	PL	UE	NP	PS	TS	MP	LP
ANB angle	1	0.210^∗^	0.462^∗^	-0.117^∗^	0.206^∗^	0.057	0.113	-0.382^∗^	-0.400^∗^	-0.415^∗^	-0.117^∗^
C2C4-SN angle	0.210^∗^	1	0.364^∗^	-0.135^∗^	0.071	-0.087	-0.036	-0.086	0.085	0.003	0.124^∗^
Palatal angle	0.462^∗^	0.364^∗^	1	-0.072	0.061	-0.016	0.439^∗^	-0.186^∗^	-0.148^∗^	-0.184^∗^	-0.043
PW	-0.117^∗^	-0.135^∗^	-0.072	1.000	-0.047	0.099	0.055	0.049	0.035	0.061	0.057
PL	0.206^∗^	0.071	0.061	-0.047	1	-0.096	0.101	-0.376^∗^	-0.084	-0.113	0.094
UE	0.057	-0.087	-0.016	0.099	-0.096	1	-0.030	0.040	-0.072	-0.076	0.091
HH	-0.327^∗^	-0.696^∗^	-0.491^∗^	0.235^∗^	-0.054	0.032	-0.079	0.055	0.082	0.148^∗^	0.075
HV	-0.006	-0.014	-0.294^∗^	0.259^∗^	0.325^∗^	0.249^∗^	-0.091	-0.007	0.087	0.040	0.279^∗^

∗: statistically significant, *p* < 0.05.

## Data Availability

This is an original article. No any published data was available.
